# A Noncontact Method for Locating Radial Artery above Radial Styloid Process in Thermal Image

**DOI:** 10.1155/2020/4057154

**Published:** 2020-05-07

**Authors:** Xingguang Geng, Su Liu, Yitao Zhang, Jiena Hou, Shaolong Zhang, Jun Zhang, Haiying Zhang

**Affiliations:** ^1^Institute of Microelectronics of Chinese Academy of Sciences, Beijing, China; ^2^University of Chinese Academy of Sciences, Beijing, China; ^3^Beijing Key Laboratory for Next Generation RF Communication Chip Technology, Beijing, China

## Abstract

A radial artery above the radial styloid process is called GUAN and is a critical position for collecting pulse wave in traditional Chinese medicine theory. Locating GUAN is a precondition for collecting radial pulse wave. However, existing methods for locating GUAN lead to large deviations. This paper proposes a novel nontouch method for locating GUAN based on thermal imaging and image processing. This method consists of three parts: the infrared thermal imaging location imaging platform, the wrist edge contour extraction algorithm based on arbitrary angle edge recognition, and radial protrusion recognition algorithm (*x* coordinate identification algorithm of GUAN) and radial artery fitting algorithm (*y* coordinate identification algorithm of GUAN). The infrared thermal imaging positioning imaging platform is used to ensure that the wrist of the subject enters the fixed imaging area in a fixed position during each measurement and transmits the thermal imaging images carrying the image information of radial processes and radial arteries to the upper computer. Arbitrary angle edge recognition algorithm is used to extract wrist contour and radial artery edge information. The *x*-axis coordinates of the radial artery were provided by the identification algorithm, and the *y*-axis coordinates of the radial artery were provided by the fitting algorithm. Finally, the *x* and *y* coordinates determine the GUAN position. The algorithm for locating GUAN could provide repeatable and reliable *x* and *y* coordinates. The proposed method shows that relative standard deviation (RSD) of *x* distance of GUAN is less than 9.0% and RSD of *y* distance of GUAN is less than 5.0%. The proposed method could provide valid GUAN coordinates and reduce deviations of locating GUAN.

## 1. Introduction

Radial pulse waves contain rich information about the physiological condition of a body. The analysis of the radial pulse waves is regarded as an important approach to evaluate patient status in traditional Chinese medicine (TCM) [1–3]. GUAN is a segment of the radial artery above the radial styloid process and a critical position for collecting pulse wave correctly in TCM diagnosis [4–8]. However, current methods for locating GUAN, including tactile sense and pressure sensor array, could not achieve precise positioning [5, 6, 9–13]. When sizes of those single sensors are almost less than 10 mm × 10 mm, it is difficult for people to place sensitive areas of a single sensor at the center of GUAN each time by tactile sense [4, 7, 13–15]. When sizes of those single sensors are large, those sensors tend to cover CUN and CHI, which are positions near GUAN [4, 7, 13–15]. Besides, locating GUAN is not achieved by the pressure sensor arrays with a small gap (less than 1 mm) [6, 10] because sensor units in the array could be susceptible to vibration interference among them through connection materials.

To improve position accuracy and repeatability, a nontouch method based on thermal imagery and image processing for locating GUAN is proposed in this paper. This method contains two parts: a specially made thermal imaging platform and an algorithm for recognizing GUAN.

Human skin has a large absorption coefficient in the visible and near UV spectrum. The images of the radial artery in deep forearm tissue are not obtained in this spectrum [16, 17]. A thermal camera provides vital information for the nontouch method. It could not only generate an image of a part of the radial artery but also reflect the wrist outline by detecting temperature differences among skin above the radial artery, skin above other tissues, and background [18].

The algorithm for locating GUAN mainly recognizes characteristics of the radial styloid process and radial artery [19–21], and coordinates of GUAN are generated according to these characteristics and TCM theory.

In this paper, we propose a novel method for locating GUAN based on thermal imaging and image processing. The method includes three steps: (1) getting the thermal image of a wrist through the proposed thermal imaging platform; (2) extracting continuous wrist edges by edge detection algorithm; (3) obtaining *x* and *y* coordinates of GUAN by recognizing features of the radial styloid process and radial artery. The proposed method could achieve better positioning accuracy and provide reliable coordinates of GUAN.

## 2. Materials and Methods

### 2.1. Thermal Imaging Platform

**Figure 1:**
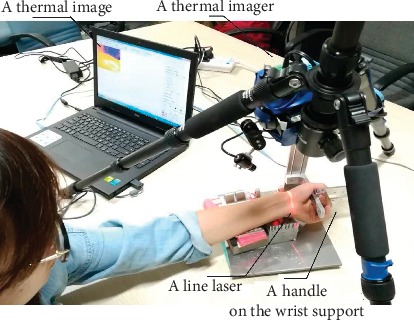
Photograph of the thermal imaging platform.

As shown in Figure 1, the proposed platform is applied to keep the wrist of the subject firmly and generate a thermal image. The platform mainly consists of the following parts: a thermal imager (MAG32, 8 to 14 *μ*m, Magnity Electronics Co. Ltd.), wrist support, and a line laser. Before imaging, the wrist crease of the subject should be aligned with a line laser. It could ensure that the wrist is placed in roughly the same position and make sure that the wrist crease is at the edge of the image. Then, the subject holds a handle tightly on the wrist support to keep a unified posture and extrude wrist joint for imaging. Finally, the thermal imager collects image information of the radial artery and the radial styloid process.

### 2.2. Edge Detection Method

The quality of edge detection is related to the detection angle, scale, and threshold. Liu et al. propose a novel edge detection algorithm to detect edges at arbitrary angles, and the optimized number of angles in the algorithm is introduced [22]. This algorithm combines singularity detection with Gaussian wavelet transform and edge detection at arbitrary directions and contains three steps:

1. A compact connection and a sparse connection are used to achieve the pixel combination principle of a corner.
2. According to the above pixel line combination principle, the image is segmented into several pixel lines along the angle, and the local maximum value of the pixel line is identified and the nonmaximum value is suppressed. The maximum point of each pixel line is recombined into an image edge along a certain angle. According to the above steps, the image edges generated by the 0° to 45° detection angle are superimposed to generate multiangle image edges.
3. According to the angle expansion rule, the detection angle from 0° to 45° is extended to 0° to 360° to generate the final image edge.

### 2.3. Locating GUAN

**Figure 2:**
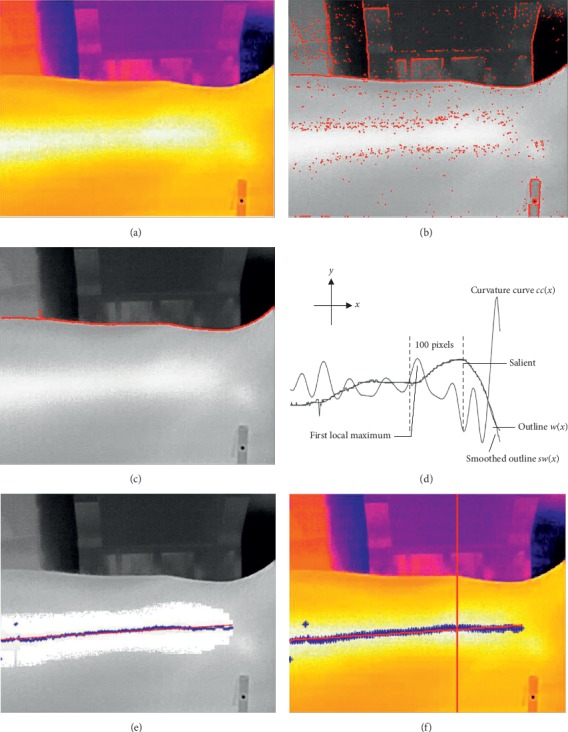
Schematic illustration of locating GUAN. (a) Thermal image of the wrist, (b) edge image of the wrist, (c) the processed edge image of the wrist, (d) schematic illustration of locating *x* coordinate of GUAN, (e) the radial artery region segmented by the grayscale features of the edge point neighborhood, and (f) thermal image marked with GUAN.

**Figure 3:**
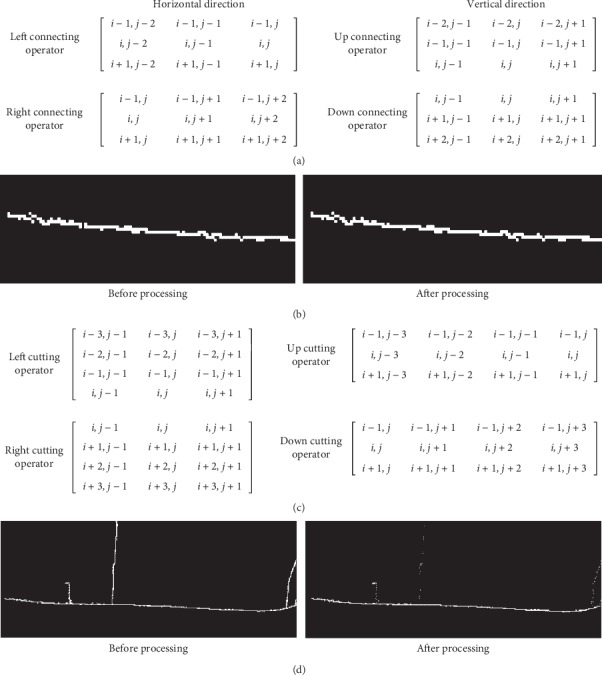
Connecting operator and cutting operator. (a) Matrix diagram of the connecting operator. (b) Effect drawing of the connecting operator. (c) Matrix diagram of the cutting operator. (d) Effect drawing of the cutting operator.

When a subject stretches his/her wrist and holds a handle on the wrist support of the proposed platform, the radial styloid process and wrist joint appear in the image (Figure 2(a)). The edges of the image (Figure 2(b)) could be obtained using an edge detection algorithm. The radial artery and the radial styloid process could be recognized based on the edges Figure 3.

#### 2.3.1. Locating *X* Coordinate of GUAN

The wrist edge could be extracted from other edges, and the radial styloid process could be recognized based on the wrist edge and its characteristics. Then, the algorithm of locating *x* coordinate of GUAN contains two parts: extracting wrist edge and locating *x* coordinate.

1. Extracting wrist edge: this preprocessing includes identifying the maximum connected domain of the wrist edge and cutting branches:
2. If the maximum connected domain reaches the boundary of the left and right sides of the image, that is, there is no break point in the connected domain, the maximum connected domain can be considered as the edge of the arm wrist. When the maximum connected domain does not reach the left and right edges of the image, it is necessary to connect the breaking point of the arm edge to form an overall edge of the arm wrist through the left and right edges of the image. The left break point of the maximum connected domain is taken as the origin to search for the edge fragment within the range of 2 pixels in the five directions of the point: up, left up, left, left down, and down. If there are other connected domains in the search area, the two connected domains will be connected, and the intermediate breakpoint pixel will supplement pixels between the two fragments by interpolation and finally form a new connected domain. Moreover, the breakpoint on the left side of the new connected domain will be taken as the origin to further search for other edge fragments until it reaches the left edge of the image. When the left edge of the maximum connected domain of the wrist is connected, the new right break point of the connected domain is taken as the origin to search and connect the edge fragments within the range of 2 pixels in the five directions of the point, up, left up, left, left down, and down, and form a new connected domain until it reaches the right edge.  Figure 3(a) shows the matrix diagram of the connecting operator. And Figure 3(b) shows the effect drawing of the connecting operator.
3. A part of the wrist edge *pw*(*x*) is extracted by identifying the maximal connected component. Then, branches of *pw*(*x*) are cut by cutting operator along the long-axis direction of *pw*(*x*). The cutting operator is chosen by a long-axis direction. When the long axis is along the *x*-axis, the horizontal cutting operator and the vertical cutting operator will cut the branches on the top left, the bottom left, the top, the bottom, the top right, and the bottom right.  Figure 3(c) shows a matrix diagram of cutting operators. And Figure 3(d) shows the effect drawing of the cutting operator. At last, the wrist edge can be extracted.
4. Locating *x* coordinate: the outline contains many steps which are caused by two row adjacent pixels and have an influence on the curvature of it. Therefore, it is smoothed through a Butterworth low-pass digital filter B where the sample frequency is 70 Hz, the passband corner frequency is 2 Hz, the stopband corner frequency is 3 Hz, passband ripple is 3 dB, and stopband attenuation is 30 dB. Then, the curvature curve *cc*(*x*) of the smoothed outline *sw*(*x*) is calculated (Figure 2(d)). Taking the salient as a landmark, the distance between recess and GUAN is less than the width of a finger (approximately 100 pixels in our platform) according to TCM theory. When *cc*(*x*_0_) is the first local maximum of *cc*(*x*)and the slope of *sw*(*x*_0_)is positive within the 100 pixels from the left of the salient, *x*_0_ is considered as *x* coordinate of GUAN. Finally, the *x* coordinate of GUAN is marked with a vertical line in the thermal image (Figure 2(d)). The main process mentioned above is described in Algorithm 1.

#### 2.3.2. Locating *Y* Coordinate of GUAN

A 10× 10 pixel region *R*(*i*, *j*) is established at each edge point in the edge image. The mean value and standard deviation of these regions are compared with the threshold values which are derived from a gray histogram of the radial artery region. As shown in Figure 2(e), when the mean value and standard deviation of the regions satisfy the threshold conditions, the pixels in the regions *RP*(*i*, *j*) are selected and used to compose an image of the partial radial artery *A*(*i*, *j*). Then, the mean values of *y* coordinate values in each column of *A*(*i*, *j*) (blue labels in Figure 2(f)) are obtained and fit an equation (red slash line in Figure 2(f)) for radial artery pattern using a least-squares method. The *y* coordinate of GUAN is obtained by the equation and the *x* coordinate of GUAN. Finally, the position of GUAN is shown at the thermal image (Figure 2(e)) and the main process mentioned above is described in Algorithm 2.

## 3. Conversion between Pixel Distance and Actual Spatial Distance

The mapping of pixel distance to space distance plays an important role in the development of 3D mobile platform of desktop pulse diagnosis instrument. The camera is placed at an angle perpendicular to the ground. Due to the upward tilt of the elbow drag, the image obtained has trapezoid distortion, that is, the object above the image (near end) is wide; the object below the image (far end) is narrow. This trapezoid distortion will cause a large error in the subsequent calculation of three-dimensional spatial coordinates, so it is unable to achieve the accurate positioning of the acquisition probe. According to the imaging principle of the camera's optical lens and other geometric relationships, the mapping relationship between the image coordinate system and the real-world coordinate system can be deduced. Only by knowing the camera's horizontal field angle, vertical field angle, the distance between the camera and the bracket, and the tilt angle of the elbow drag, the actual spatial coordinates of the guan position can be determined.

**Figure 4:**
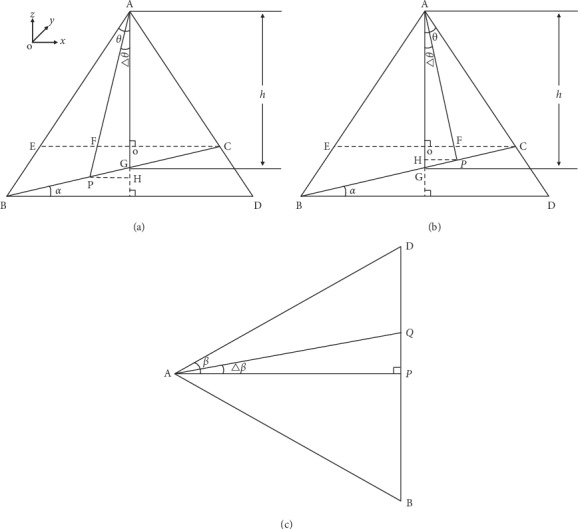
(a) Geometric side view of thermal imaging platform (Case 1). (b) Geometric side view of thermal imaging platform (Case 2). (c) Top view of thermal imaging platform.

Assume that point P is the point location where the coordinate needs to be determined, and point O is the reference origin. Set the pixel position corresponding to P point as (*x*, *y*). Because of the upward inclination of the elbow drag, the calculation of the mapping value of the actual spatial coordinate of point P in the *x*-axis can be divided into two cases Figure 4.

  Case 1: as shown in Figure 4(a), point P is assumed to be on the left side of point G. Then, the step angle of point P can be expressed by the following formula:

(1) $$\begin{array}{c} \Delta \theta =\left|\frac{X}{\text{height}/2}\theta \right|. \end{array}$$

  In formula (1), *θ* is half of the vertical field angle. Height is the height of the image. According to the sine theorem, the following formula can be obtained:

(2) $$\begin{array}{c} \frac{\text{GP}}{\sin\left(\Delta \theta \right)}=\frac{h}{\sin\left(90-\Delta \theta -\alpha \right)}, \end{array}$$

(3) $$\begin{array}{c} \frac{\text{AP}}{\sin\left(90+\alpha \right)}=\frac{\text{GP}}{\sin\left(\Delta \theta \right)}. \end{array}$$

  In formula (2), *α*  is the tilt angle of the bracket, *h* is the distance between the camera and the bracket (perpendicular to the horizontal plane, and the intersection point with the horizontal plane is o point). Through the formula transformation, we can get the distance HP and the length of AP between the mapping point of point P in the *x*-axis and the distance O.

(4) $$\begin{array}{c} \text{HP}=\frac{\sin\left(\Delta \theta \right)*h}{\sin\left(90-\Delta \theta -\alpha \right)}*\sin\left(90-\alpha \right),\\ \text{AP}=\frac{\sin\left(90+\alpha \right)*h}{\sin\left(90-\Delta \theta -\alpha \right)}. \end{array}$$

  According to the coordinate system direction, we can see the *x*-axis coordinates of point P as follows:

(5) $$\begin{array}{c} x_{P}=-\text{HP.} \end{array}$$

  Case 2: as shown in Figure 4(b), point P is assumed to be on the right side of point G. According to the sine theorem, the following formula can be obtained:

(6) $$\begin{array}{c} \frac{\text{GP}}{\sin\left(\Delta \theta \right)}=\frac{h}{\sin\left(90-\Delta \theta +\alpha \right)},\\ \frac{\text{AP}}{\sin\left(90-\alpha \right)}=\frac{h}{\sin\left(90-\Delta \theta +\alpha \right)},\\ \text{HP}=\frac{\sin\left(\Delta \theta \right)*h}{\sin\left(90-\Delta \theta +\alpha \right)}*\sin\left(90-\alpha \right),\\ \text{AP}=\frac{\sin\left(90-\alpha \right)*h}{\sin\left(90-\Delta \theta +\alpha \right)}. \end{array}$$

According to the coordinate system direction, we can see the *x*-axis coordinates of point P as follows:

(7) $$\begin{array}{c} x_{\text{P}}=\text{HP.} \end{array}$$

As shown in Figure 4(c), point Q is the mapping point of point P on the *y*-axis.

Then, the step angle of point Q can be expressed by the following formula:

(8) $$\begin{array}{c} \Delta \beta =\left|\frac{Y}{\left(width|/|2\right)}\beta \right|. \end{array}$$

According to the sine theorem,

(9) $$\begin{array}{c} \text{PQ}=\text{AP}*\tan\left(\Delta \beta \right). \end{array}$$

According to the coordinate system direction, we can see the *y*-axis coordinates of point P as follows:

(10) $$\begin{array}{c} y_{\text{P}}=\text{PQ}*\text{sign}\left(Y\right). \end{array}$$

In formula (10), sign is a sign function.

## 4. Experiments and Results

**Figure 5:**
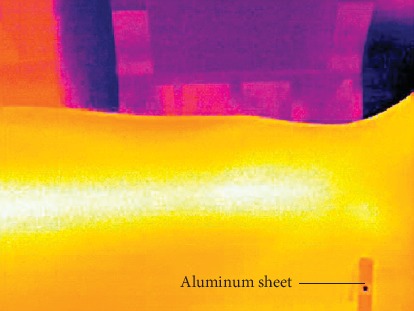
Experimental diagram of closing position.

During the location process, each subject is required to hold the handle on the wrist support and removes the wrist after finishing image acquisition. An aluminum sheet with thermal insulation material (1 mm × 1 mm) is pasted on the wrist crease of a subject and is considered as a reference coordinate to eliminate position deviation (a thermal image with the aluminum sheet is shown in Figure 5). The process is repeated ten times and each subject is required to keep a unified posture. Eight volunteers (25.4 ± 2.1 years, 169.3 ± 6.4 cm, 59.4 ± 11.2 kg; 5 males and 3 females) from our lab participated in the experiment.

Table 1 shows *x* distance between GUAN and wrist crease (*x* coordinate of wrist crease − *x* coordinate of GUAN) and *y* distance of GUAN (*y* coordinate of GUAN). The standard deviations of *x* distance are all less than 1.7 mm. The standard deviations of *y* distance are almost less than 1 mm. The RSD of *x* distance is less than 9.0% and the RSD of *y* is less than 5.0%.

## 5. Discussion

### 5.1. The Effect of Hand Posture and Position

In this paper, an imaging platform based on thermal imager is designed for imaging radial processes and radial arteries. In this paper, the closed position imaging platform is used to maintain the wrist state of the subject and generate thermal imaging images. The imaging platform is mainly composed of the thermal imager, wrist bracket, a line laser, and a host computer. A line laser can ensure that the wrist is placed in roughly the same position and make sure that the wrist crease is at the edge of the image. Wrist branches can highlight the radial artery of the wrist. However, when placing the arm, the crease lines on the wrist should be included in the image. Meanwhile, the height of the arm should be slightly lower than that of the wrist. This can further ensure that the high posterior metacarpal bone presents a complete envelope.

### 5.2. The Effect of Location of GUAN

The *x* distance between wrist crease and GUAN is introduced for evaluating the accuracy of the *x* coordinate of GUAN. We set a reference point (the aluminum sheet) in order to eliminate position deviation and investigate deviations caused by other factors because position deviation has little influence on the guiding sensor.

Table 1 shows that *x* distance of GUAN is fairly reproducible (RSD< 9.0% and standard deviation < 1.7 mm). The deviation could be mainly due to wrist shape changes and could not be eliminated. However, it could be accepted because it could be covered by the sensitive area of a single sensor and it is also smaller than the spatial resolution of a sensor array. *Y* distance of GUAN is also fairly reproducible (RSD< 5.0% and standard deviation < 1.5 mm). The standard deviation of *y* distance is effective in locating *y* coordinate of GUAN for a single sensor or a sensor array. Furthermore, most of the deviation of *y* distance is smaller than the radial artery diameter. Therefore, the deviation of *y* distance provides valid values for guiding sensor. Conclusively, the proposed location algorithm is effective and necessary for locating GUAN.

### 5.3. The Limitation of This Study

In this paper, only eight young people are used to verify the accuracy of the system. With the growth of age and the loss of human collagen, blood vessels are more convex, so the GUAN position of the wrist of the elderly is more easily detected, so it is not considered. With high BMI subject, it is usually more difficult to discern the radial artery pulsation and styloid process. The detection accuracy of the system is affected by the accuracy of the infrared camera itself. However, the higher the accuracy, the higher the price of the infrared camera itself. For this defect, we will make the results in the manufacture of the trolley-type pulse diagnosis instrument. Meanwhile, in future work, we will improve the proposed location algorithm to decrease the standard deviations of *x* and *y* coordinates of GUAN.

## 6. Further Work

In future work, we will improve the proposed location algorithm and buy high-precision infrared thermal imaging equipment to decrease the standard deviations of *x* and *y* coordinates of GUAN. In addition, the wrist support, the thermal imager, and pressure sensors will be integrated together to achieve the real-time location of GUAN and the collection of the radial pulse waves.

## 7. Conclusions

In this paper, a novel method for locating GUAN based on thermal imaging and image processing is proposed. The infrared thermal imaging positioning imaging platform is used to ensure that the wrist of the subject enters the fixed imaging area in a fixed position during each measurement and transmits the thermal imaging images carrying the image information of radial processes and radial arteries to the upper computer. Arbitrary angle edge recognition algorithm is used to extract wrist contour and radial artery edge information. The *x*-axis coordinates of the radial artery were provided by the identification algorithm, and the *y*-axis coordinates of the radial artery were provided by the fitting algorithm. Finally, the *x* and *y* coordinates determine the GUAN position. The results of locating GUAN show that the proposed location algorithm not only has better repeatability but also provides a valid reference for collecting pulse waves of GUAN. Finally, the novel method could provide valid GUAN coordinates.

## Figures and Tables

**Algorithm 1:**
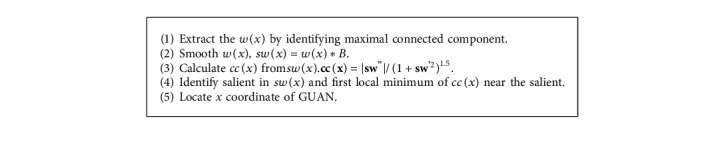
Algorithm of locating *x* coordinate of GUAN.

**Algorithm 2:**
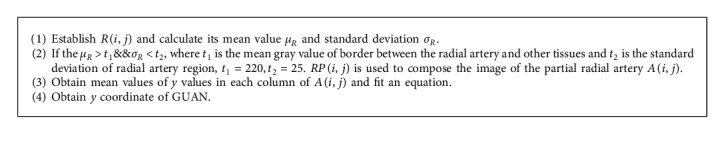
Algorithm of locating *y* coordinate of GUAN.

**Table 1:** Results of location of GUAN.

Subject	*X* distance (mm)			*Y* distance (mm)		
	Average	Standard deviation	RSD	Average	Standard deviation	RSD
1	19.55	1.41	0.072	33.14	0.25	0.008
2	14.44	0.41	0.028	31.93	0.19	0.006
3	15.81	1.09	0.069	32.62	0.50	0.015
4	19.53	1.65	0.084	36.90	0.46	0.012
5	26.03	1.56	0.060	30.52	1.47	0.048
6	16.91	1.35	0.080	26.54	0.63	0.024
7	20.31	1.27	0.063	35.46	1.07	0.030
8	24.58	1.45	0.059	32.42	0.45	0.014

## Data Availability

The raw/processed data required to reproduce these findings cannot be shared at this time as the data also form part of an ongoing study. But in the near future, with the permission of the institution, data sharing will be considered.
